# Electrochemical fractionation of stable sulfur isotopes in a rechargeable lithium–sulfur battery: a revisit from the law of mass conservation

**DOI:** 10.1039/d5sc10219g

**Published:** 2026-02-14

**Authors:** Yu-Hui Zhu, Sen Xin

**Affiliations:** a Laboratory of Molecular Nanostructure and Nanotechnology, CAS Research/Education Center for Excellence in Molecular Sciences, Beijing National Laboratory for Molecular Sciences (BNLMS), Institute of Chemistry, Chinese Academy of Sciences (CAS) Beijing 100190 P. R. China zhuyuhui@iccas.ac.cn xinsen08@iccas.ac.cn; b School of Chemical Sciences, University of Chinese Academy of Sciences Beijing 100049 P. R. China

## Abstract

Fractionation of stable ^32^S/^34^S isotopes occurs *via* a ‘lithium polysulfide (LiPS) shuttle’ process upon (dis)charging a Li–S battery, with lighter ^32^S isotope species with a larger diffusion coefficient enriched at the anode side. However, the global distribution of S isotopes within the battery and its dependence on the state of charge (SoC) of battery remain unclear. In this work, we quantitatively measured the S isotope distribution in the cathode, anode, and electrolyte of a Li–S battery at different SoCs using triple-quadrupole inductively coupled plasma mass spectrometry (TQ-ICP-MS). By establishing a unified sample pretreatment protocol for all S species, we demonstrate, for the first time, mass conservation of S isotopes within a cycled Li–S battery. Loss of active ^32^S species from the cathode to the electrolyte (and finally to the anode) accounts for enrichment of ^34^S species at the cathode during battery cycling. Based on isotope mass conservation, the separation factors of ^32^S and ^34^S were found to be positively correlated, while trade-offs were found both between the single-stage yields of the two isotopes and between the separation factor and yield of a given isotope. Our findings help define the boundary conditions for key parameters (*e.g.*, separation factors, yields and stages) for electrochemical cascade separation of stable S isotopes.

## Abstract 2

Mass conservation of stable S isotopes was verified in a cycled Li–S battery: ^32^S is enriched at the anode and in the electrolyte with a high separation factor (*α*) and a low yield (*Y*), while ^34^S is enriched at the cathode with a low *α* and a high *Y*.

## Graphical abstract

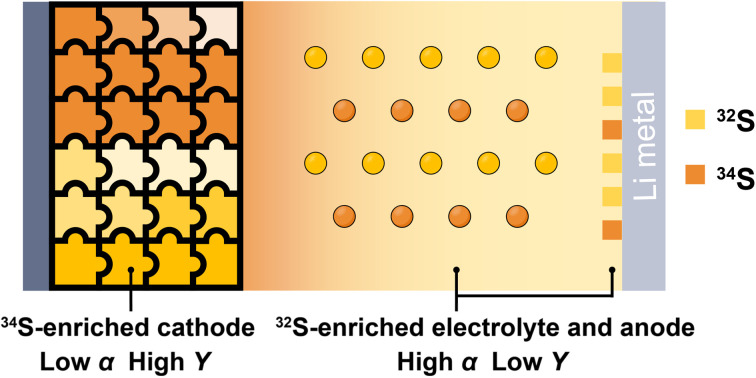

## Introduction

**Fig. 1:**
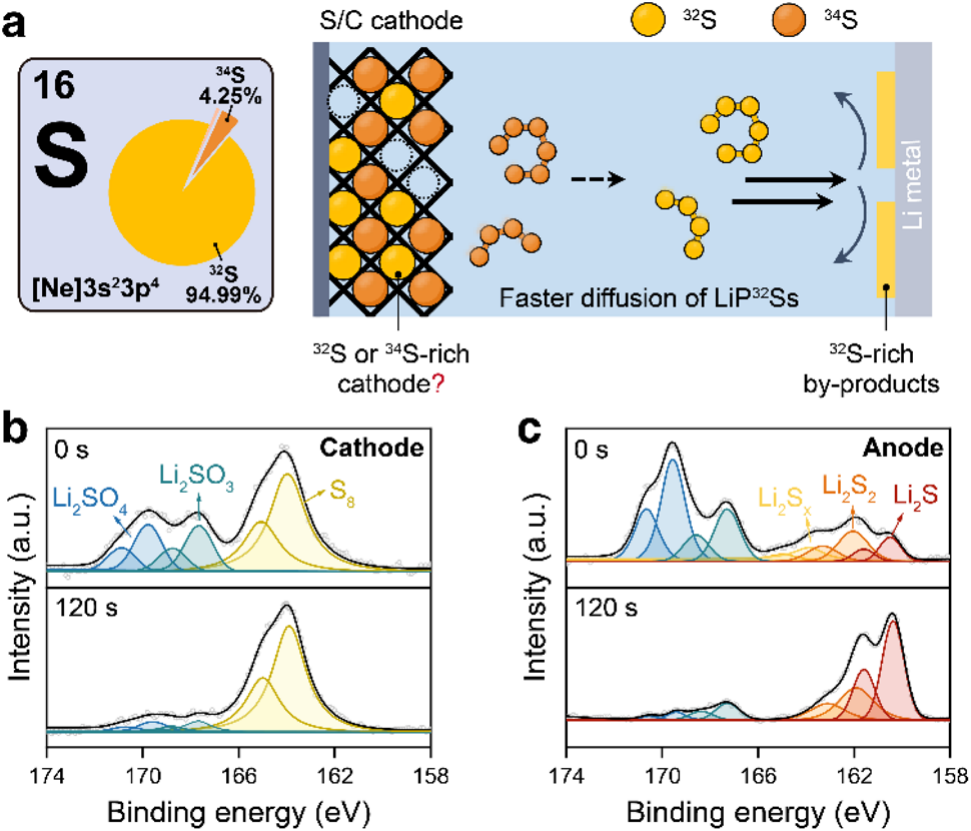
(a) Schematic illustration of the stable S isotope fractionation in a Li–S battery during a battery discharge and charge process. S 2p XPS spectra of (b) the cathode and (c) the anode of a Li–S battery after a single discharge–charge cycle.

Lithium–sulfur (Li–S) batteries are promising high-energy rechargeable batteries owing to their high theoretical capacities of both the cathode (1675 mAh g^−1^) and the anode (3860 mAh g^−1^), offering potential for emerging applications including long-range electric vehicles and low-altitude aviation.^1–4^ The electrochemistry of the S cathode during battery (dis)charge involves a complex two-electron conversion reaction occurring at the solid–liquid (S-electrolyte or Li_2_S-electrolyte) interface, where soluble lithium polysulfides (LiPSs, *i.e.* Li_2_S_*x*_, 4 ≤ *x* ≤ 8) form as intermediates between the terminal products S and Li_2_S.^5–8^ As LiPSs are soluble in the liquid ether electrolyte, they tend to ‘shuttle’ from the cathode to the Li-metal anode and be reduced at the anode, causing irreversible loss of cathode S and passivation of the anode surface.^9–12^ Recently, the kinetic isotope effect of S has been identified in the LiPS shuttle process, where the differences in diffusivities of isotopic LiPSs (*e.g.* Li_2_^32^S_6_ and Li_2_^34^S_6_) induce significant S isotope fractionation in the S-containing byproducts at the anode (Fig. 1a).^13,14^ Time-of-flight secondary ion mass spectrometry (ToF-SIMS) measurements revealed a remarkably high ^32^S/^34^S separation factor (*α* = 1.6 to 2.0, Note S1), one to two orders of magnitude higher than those reported from the conventional isotope separation techniques.^15–17^ These findings indicate a potentially low-cost and energy-efficient S isotope separation method by taking advantage of the electrochemical shuttle process.

Despite the intriguing results, several critical problems remain unresolved. As a localized and surface-sensitive method, ToF-SIMS cannot provide a complete picture of isotopic S distribution at the anode.^18,19^ Specifically, ToF-SIMS analysis in two different sampling regions of the same cycled Li anode revealed slightly different *α* values and inconsistent surface layer thicknesses (Fig. S1). These discrepancies demonstrate that the high spatial sensitivity of ToF-SIMS captures localized fluctuations rather than the integrated isotopic distribution. In the case of local inhomogeneity, the isotope ratios measured by ToF-SIMS exhibit variations within a certain range and thus cannot provide a fully accurate picture of the entire electrode, despite showing a consistent overall trend.

To substantiate the high separation factor and exclude any potential experimental artefact, it is essential to verify the S isotope mass conservation within the battery; that is, ^32^S and ^34^S are spatially separated at different components of the Li–S battery, and the total abundance of S isotopes in the cycled cathode, anode and electrolyte must remain consistent with that of the pristine S cathode. More importantly, the law of isotopic mass conservation fundamentally correlates the separation factors and yields of ^32^S and ^34^S across various battery components, thereby establishing the theoretical foundation for practical isotope separation applications. In this work, we develop a standardized protocol for quantitative S isotope analysis in Li–S batteries using triple-quadrupole inductively coupled plasma mass spectrometry (TQ-ICP-MS). Given the diversified chemical forms of S in different cell components (S^2−^, S_*x*_^2−^, S, SO_4_^2−^*etc.*), all S species are uniformly converted to sulfate prior to the ICP-MS measurements. Using this approach, we accurately quantified the abundance of ^32^S and ^34^S in the cycled Li anode, electrolyte, and composite S cathode, and for the first time, confirmed the mass conservation of S isotopes within the Li–S battery. These findings verify the substantial occurrence of electrochemical S isotope fractionation with high separation factors at both electrodes of the cycled Li–S battery and demonstrate the practical viability of stable S isotope separation *via* an electrode reaction.

## Results and discussion

The electrochemical measurements were performed using Swagelok-type cells assembled with a composite S/C electrode cathode and a Li metal anode. The composite S/C material was prepared *via* a melting-diffusion method by mixing equimolar ^32^S and ^34^S with ordered mesoporous carbon (CMK-3). The S content in the composite was controlled at approximately 40% according to thermogravimetric results (Fig. S2). The chemical compositions of S species in the cycled cathode and Li anode were first examined by X-ray photoelectron spectroscopy (XPS) to develop appropriate sample preparation protocols. As shown in Fig. 1b and c, after a single discharge–charge cycle, the cathode is dominated by elemental S_8_, whereas the Li metal surface is primarily built up by reduced species, including Li_2_S, Li_2_S_2_, and LiPSs. Being discharged to 1.5 V, the cathode surface contains mainly Li_2_S_2_ and low-order LiPSs, while the anode surface contains mainly Li_2_S and Li_2_S_2_ (Fig. S3). In addition, oxidized S products such as Li_2_SO_3_ and Li_2_SO_4_ are detected on the outermost surfaces of both electrodes at different states of charge (SoCs). *In situ* Raman spectroscopy reveals that S species in the electrolyte exist predominantly as LiPSs with varied chain lengths throughout the discharge–charge cycle (Fig. S4). Generally, these observations indicate that S in a cycled Li–S battery primarily presents as reduced sulfides at the anode, as soluble LiPSs in the electrolyte and as either S_8_ or Li_2_S_2_ at the cathode, depending on the state of charge.^20–22^

**Fig. 2:**
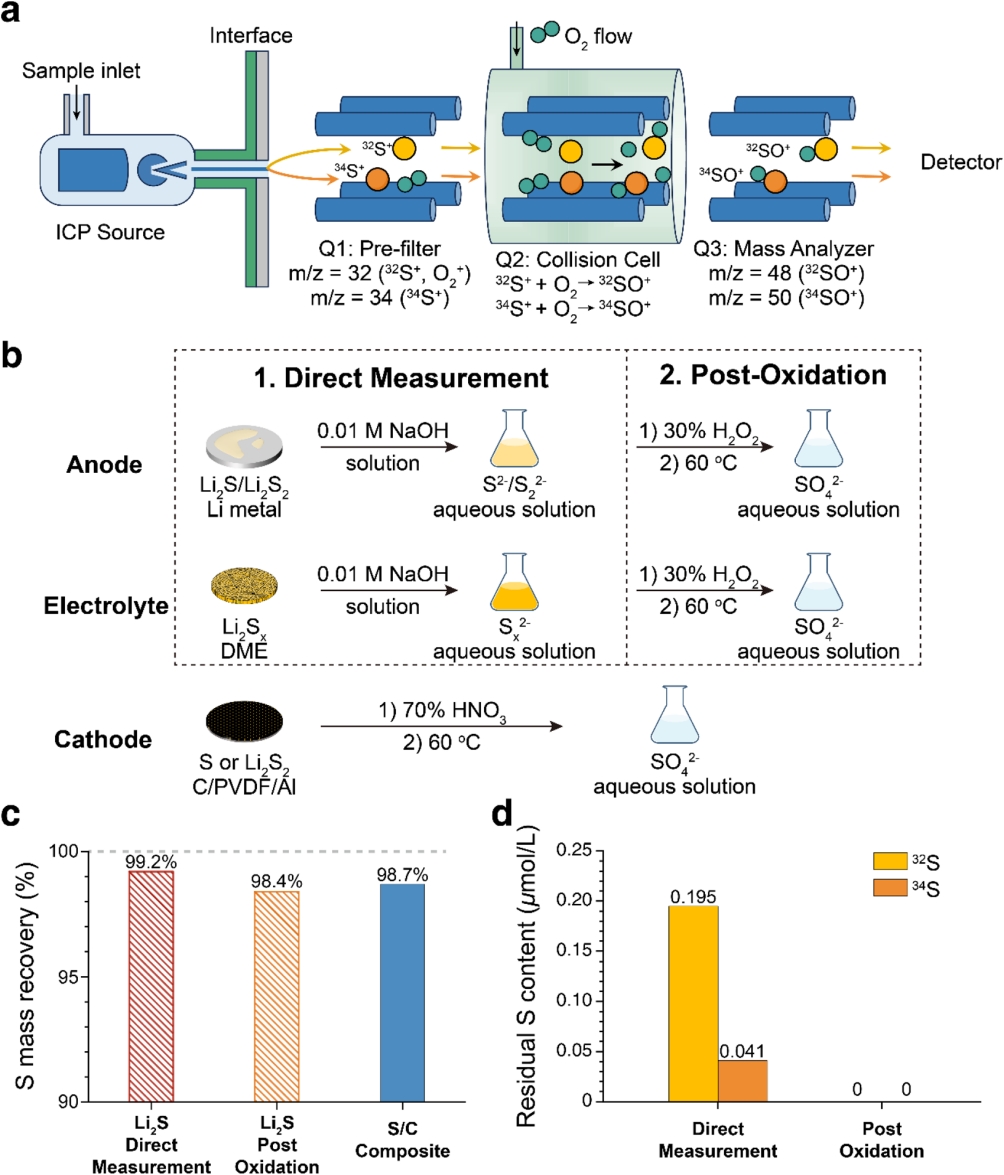
(a) Schematic illustration showing the working principle for analyzing S isotopes using TQ-ICP-MS. (b) Sample preparation methods for analyzing S isotopes in the anode, electrolyte and cathode of a Li–S battery. (c) Total S mass recovery of Li_2_S and S/C composites using the sample preparation methods in (b) by TQ-ICP-MS tests. (d) Residual S signals of the deionized water following the test for Li_2_S using the direct measurement and post-oxidation methods, respectively.

Triple-quadrupole inductively coupled plasma mass spectrometry (TQ-ICP-MS) was employed to quantify both absolute S content and isotopic compositions of the S-containing species in different battery components. Compared with the conventional single-quadrupole ICP-MS which suffers from severe isobaric and polyatomic interferences in S isotope analysis (Table S1) and multi-collector ICP-MS that requires extensive sample purification and a high instrumental cost, TQ-ICP-MS shows advantages in terms of high sensitivity, robust interference removal, and minimal sample preparation requirements.^23–25^ The principle for S isotope analysis using TQ-ICP-MS is illustrated in Fig. 2a. In this technique, aqueous solutions are aerosolized using a nebulizer and then atomized in the plasma torch before entering the first quadrupole (Q1) for initial mass filtering. In Q1, ions with mass-to-charge ratios (*m*/*z*) of 32 and 34 are selected, and the other interfering ions with *m*/*z* of 48 and 50 are filtered. ^32^S^+^, ^34^S^+^ and interferents (mainly ^16^O_2_^+^) are transmitted into an oxygen-filled collision cell (Q2), where S^+^ is selectively converted into SO^+^ species (*i.e.*^32^SO^+^ and ^34^SO^+^). The third quadrupole (Q3) then performs the final mass discrimination at *m*/*z* values of 48 and 50, which helps to eliminate O_2_^+^ and other interfering species and enable interference-free acquisition of ^32^S and ^34^S abundances and isotope ratios.

Prior to the TQ-ICP-MS analysis, S species in the anode, electrolyte and cathode were converted into aqueous solutions. Because elemental S is insoluble in water, the composite S/C cathode was treated with 70% HNO_3_ and heated at 60 °C to ensure complete oxidation of S_8_ to SO_4_^2−^.^26^ Two sample-preparation strategies were evaluated for S analysis of the anode and the electrolyte (Fig. 2b): (i) direct measurement after dissolution of S-containing species in an aqueous solution or (ii) post-oxidation treatment in which all S species were converted to SO_4_^2−^ prior to the measurement. For the direct measurement method, the cycled Li anode and electrolyte were directly dissolved in 0.01 M NaOH aqueous solution. The alkaline environment effectively suppresses hydrolysis of S^2−^ and retain S in the forms of HS^−^ and HS_*x*_^−^, so that it helps to minimize S loss as H_2_S gas (Fig. S5). For the post-oxidation method, an additional step was introduced in which 30% H_2_O_2_ was added to the alkaline solution to fully oxidize S^2−^ and S_*x*_^2−^ to SO_4_^2−^.^27^ After completion of the oxidation, the solutions were diluted to a fixed volume and concentration to eliminate the matrix effect for accurate ICP-MS analysis.

Key TQ-ICP-MS parameters for ^32^S and ^34^S measurements are summarized in Table S2. The calibration curves for ^32^S and ^34^S obtained in this study are shown in Fig. S6. Both isotopes exhibit high linearity across a broad concentration range (*ca.* 3.0 µmol L^−1^ to 300 µmol L^−1^ for ^32^S and *ca.* 0.13 µmol L^−1^ to 13 µmol L^−1^ for ^34^S). To validate the accuracy of the proposed protocol for S isotope quantification in Li–S batteries, Li_2_S and S/C composites were employed as reference materials. Two sample-preparation strategies (direct measurement and post-oxidation treatment) were evaluated, whose TQ-ICP-MS results were compared with the known reference values. For both preparation methods, the measured S contents of Li_2_S and S/C composites closely match the reference values, confirming the reliability of the analytical procedures (Fig. 2c and Table S3). Notably, severe S memory effects were observed for the direct measurement method of Li_2_S, as evidenced by substantial residual S signals detected in subsequent blank solution test (Fig. 2d and Table S4). At relatively low S concentrations of 4.583 µmol L^−1^ for ^32^S and 0.21 µmol L^−1^ for ^34^S, the residual S concentrations obtained from the subsequent blank test are 0.195 and 0.041 µmol L^−1^, which correspond to 4.25% and 19.5% of the original signals, respectively. In contrast, there is no detectable S residue in the blank solutions following post-oxidation treatment, even after analyzing samples with a notably higher S concentration of *ca.* 22 µmol L^−1^. The residual S signals in the direct measurement method mainly originate from partial hydrolysis of S^2−^ and S_*x*_^2−^ species under neutral or mildly acidic conditions, which could account for formation of H_2_S gas. H_2_S is retained within the ICP-MS system, where it continuously influences subsequent measurements and generates background signals of S. To ensure the accuracy and reproducibility, the post-oxidation sample-preparation method was employed for quantifying S isotopes in Li–S batteries.

**Fig. 3:**
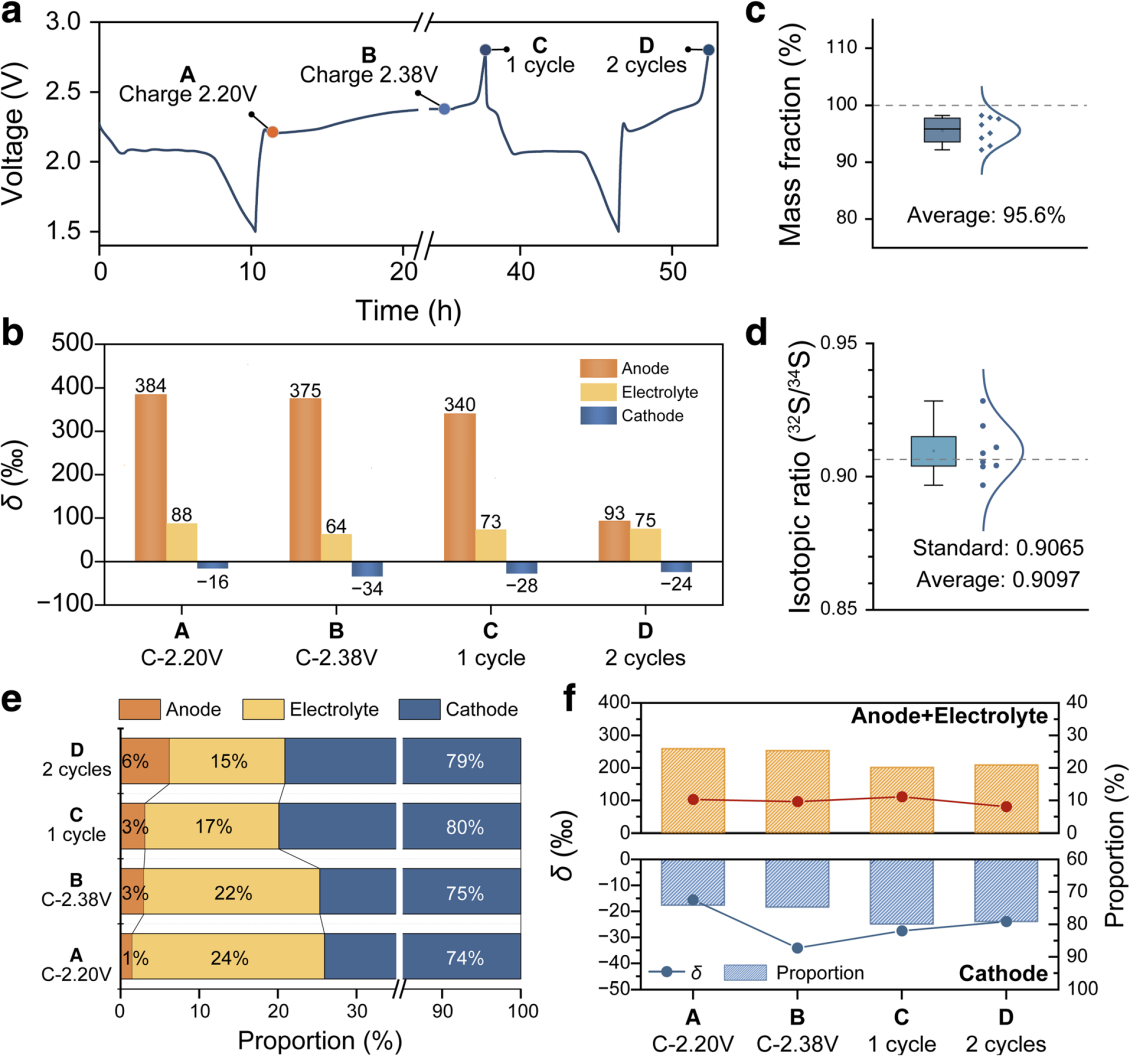
(a) Galvanostatic discharge–charge voltage profiles of the Li–S battery for isotope separation. (b) Separation factors (in the *δ* form) for the anode, electrolyte and cathode of the Li–S battery at different SoCs. (c) Statistical distribution of total S recovery and (d) overall isotopic ratios of ^32^S/^34^S from the anode, electrolyte and cathode of a cycled Li–S battery determined by TQ-ICP-MS. (e) S mass contents collected from in the anode, the electrolyte and the cathode under different SoCs. (f) Evolution of S mass contents and separation factors at the anode/electrolyte and the cathode at different SoCs.

The mass conservation of S isotopes in cycled Li–S batteries was then examined. A representative discharge–charge profile of the Li–S battery for isotope separation experiments is shown in Fig. 3a. Li–S batteries at different SoCs (*i.e.*, charged to 2.20 V, charged to 2.38 V, 1 cycle, and 2 cycles) were selected to determine the evolution of separation factors among the battery components (*i.e.*, anode, electrolyte and cathode) and to verify S isotope mass conservation. The separation factors derived from TQ-ICP-MS analysis for each battery component are summarized in Fig. 3b and Tables S5–S7. Across all electrochemical states, ^32^S is enriched at the anode and in the electrolyte, whereas ^34^S preferentially accumulates at the cathode. Statistical analysis of parallel measurements indicates that total S recovery from all battery components accounts for an average value of 95.6% of the initial S mass, demonstrating that nearly all S is successfully recovered using the new sample preparation method (Fig. 3c). The minor loss of S is likely attributed to residual electrolyte during cell disassembly. Importantly, the overall S isotope ratio obtained by summing all components of the battery, which is 0.9097 on average, closely matches that of the pristine cathode (0.9065) (Fig. 3d), which strongly proves the S isotope mass conservation in the battery (Table S8). Furthermore, we are able to quantitatively determine the S content in each component of the Li–S battery under different electrochemical states (Fig. 3e). In all cases, the cathode contains the majority of total S, which is typically more than 70% in the battery. The electrolyte generally contains 15 to 25% of the total S inventory, while the anode contains less than 10%. In addition, S at the cathode shows relatively low separation factors (*δ* = −16 to −34‰), while S at the anode shows notably higher separation factors (*ca.* 340 to 384‰ in the first cycle).

From a theoretical perspective, the efficiency of an isotope separation is primarily dominated by the isotope separation factor (*α*) and the single-stage yield (*Y*). Electrochemical S isotope separation involves selective recovery of ^34^S from the cathode and ^32^S from the anode and electrolyte. The evolution of S mass contents and separation factors at the cathode and at anode/electrolyte under different electrochemical states is summarized in Fig. 3f. For cathode-side enrichment of ^34^S, charging the battery to 2.38 V affords the highest separation factor (*δ* = −34‰) and a relatively lower single-stage yield (*Y* = −75‰). In contrast, enrichment of ^32^S at the anode and in the electrolyte exhibits an almost constant separation efficiency across various electrochemical states.

**Fig. 4:**
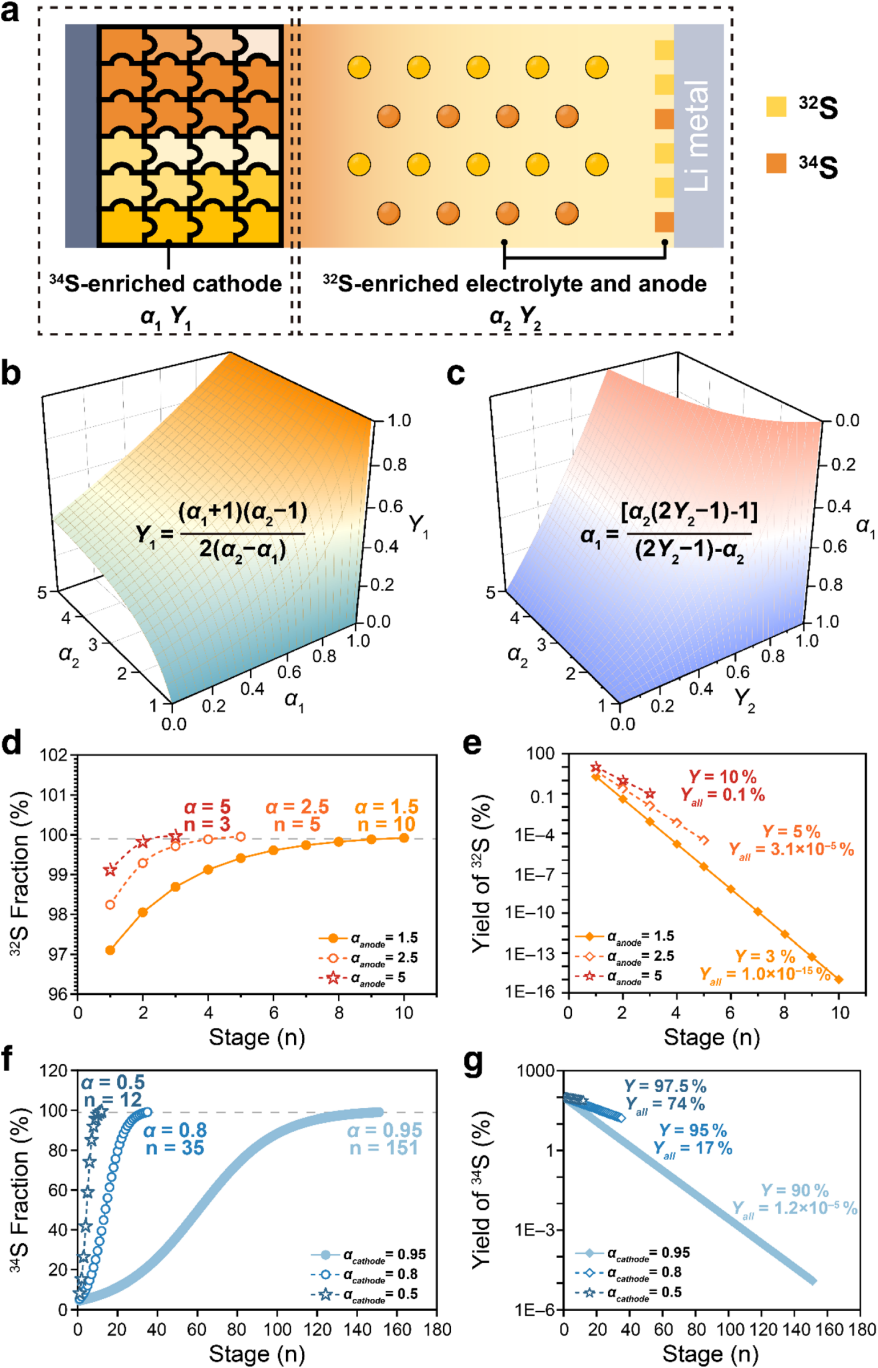
(a) Schematic diagram showing the isotopic mass conservation in a Li–S battery. (b) The 3D surface plot of the relationship between the ^34^S yield (*Y*_1_) and separation factors of ^32^S (*α*_2_) and ^34^S (*α*_1_). (c) The 3D surface plot of the relationship between the ^34^S separation factor (*α*_1_) and ^32^S separation factor (*α*_2_) and yield (*Y*_2_). (d) Correlations between separation stages and the ^32^S fraction at the anode side under the separation factors of 1.5, 2.5 and 5. (e) Correlations between the separation stages and total yields of ^32^S. (f) Correlations between separation stages and ^34^S fraction at the cathode side under the separation factors of 0.95, 0.8 and 0.5. (g) Correlations between the separation stages and total yields of ^34^S.

Constrained by the law of isotopic mass conservation, the total inventory of ^32^S and ^34^S across all battery components remains constant throughout the cycling process. Specifically, the increment of ^32^S in the anode and electrolyte after cycling is counterbalanced by the enrichment of ^34^S (or the corresponding depletion of ^32^S in the cathode). Consequently, the separation factors and yields for ^32^S and those for ^34^S in various battery components are intrinsically coupled and fundamentally governed by the law of S isotope mass conservation. To elucidate these intrinsic relationships, we further construct a quantitative model based on S isotope mass conservation (Fig. 4a). For clarity, the ratio of ^32^S to ^34^S in the pristine cathode prior to cycling is assumed to be 1 : 1. Under this assumption, the separation factors and single-stage yields for ^34^S (*α*_1_, *Y*_1_, cathode) and ^32^S (*α*_2_, *Y*_2_, anode and electrolyte) are constrained by S isotope mass conservation (Note S2):

1 *Y*_1_ + *Y*_2_ = 1

2 *Y*_1_ = (*α*_1_ + 1)(*α*_2_ − 1)/[2(*α*_2_ − *α*_1_)]

3 *α*_1_ = [*α*_2_(2*Y*_2_ − 1) − 1]/[(2*Y*_2_ − 1) − *α*_2_]

where *α*_1_ and *α*_2_ refer to the separation factors of ^34^S from the cathode and ^32^S from the anode and electrolyte, respectively; *Y*_1_ and *Y*_2_ denote the molar ratios of S in the cathode and in the anode and electrolyte relative to total S in the battery. It should be noted that the cathode side is enriched in ^34^S, resulting in 0 < *α*_1_ < 1. In this context, a higher degree of ^34^S enrichment corresponds to a lower value of *α*_1_.

Eqn (1) follows directly from mass conservation of S, namely that the total S content remains unchanged before and after cell cycling. Eqn (2) and (3) can be derived by further invoking isotopic conservation, whereby the total amounts of ^32^S and ^34^S are individually preserved during cycling. These relationships indicate that the separation efficiencies of ^32^S and ^34^S are intrinsically coupled. Specifically, the separation factor of one isotope is determined by both the separation factor and the yield of the other, and the single-stage yield can be expressed as explicit functions of the separation factors within the cell.

To more intuitively illustrate the relationship, three-dimensional (3D) plots of eqn (2) and (3) were constructed as shown in Fig. 4b and c, respectively. According to Fig. 4b, larger values of *α*_1_ and *α*_2_ lead to a higher *Y*_1_. At a fixed *α*_2_, a clear trade-off has been observed between the yield and the separation factor of ^34^S: a higher degree of ^34^S enrichment (lower *α*_1_) results in a lower yield (Fig. S7a). By contrast, when the ^34^S separation factor is held constant, the yield of ^34^S increases rapidly with increasing ^32^S separation factor and then approaches a plateau (Fig. S7b). According to Fig. 4c and S7c and d, increasing either the separation factor or the yield of ^32^S is beneficial for the enrichment of ^34^S (*i*.*e*., a decrease in *α*_1_).

Moreover, under the constraint of isotopic mass conservation, the separation factor and yield for ^34^S enrichment at the cathode side can only assume values within a bounded region, and they cannot both be increased arbitrarily or independently. The same limitation applies to ^32^S separation at the anode and electrolyte. As shown in Fig. S8a and b, the admissible ranges of the separation factor and yield for the cathode and anode are given by the projections of Fig. 4b and c onto the *α*_2_ and *α*_1_ axes, respectively. Mathematical expressions for these bounds are provided in Note S3.

Based on the above analysis, the following conclusions can be drawn:

(i) The yields of ^32^S and ^34^S are inherently competitive.

(ii) For ^34^S (and likewise for ^32^S), a fundamental trade-off exists between achieving an ultra-high separation factor and maximizing yield, requiring balanced optimization of both parameters to achieve optimal separation performance.

(iii) Enhancing both the yield and the separation factor of ^32^S facilitates the enrichment of ^34^S (*i.e.*, a reduction in *α*_1_).

Regarding the practical application of isotope separation, another paramount factor is the overall yield (*Y*_all_), which dictates the total cost of the separation and recovery process. The overall yield (*Y*_all_) is determined by the product of the stage number and the single-stage yield.

4 *Y*_all_ = *Y*^*n*^

For the separation of ^32^S, as the separation factor at the anode is significantly higher than that in the electrolyte, we here focused solely on the separation of ^32^S at the anode side and neglected the influence from the electrolyte. Starting from natural S (^32^S/^34^S = 22.35) and assuming a constant separation factor per stage, the number of separation stages (*n*) required to reach a target isotope abundance (*A*) is dictated by the separation factor:

5 *A*(^32^S) = 22.35 × *α*_anode_^*n*^/(1 + 22.35 × *α*_anode_^*n*^)

6 *A*(^34^S) = 1/(1 + 22.35 × *α*_cathode_^*n*^)

We first demonstrated the influence of different separation parameters on the separation efficiency. As shown in Fig. 4d, increasing the separation factor from the current value of 1.5 to 2.5 and 5 for ^32^S would reduce the separation stages from 10 to 5 and 3, respectively. Accompanied by improvements in the single-stage yield from 3% to 5% and 10%, the total yield of ^32^S would increase dramatically from 1.0 × 10^−15^% to 3.1 × 10^−5^% and 0.1% (Fig. 4e). At the cathode side, high-purity ^34^S (>99%) currently requires approximately 151 separation stages at *α* = 0.95, yielding an overall recovery of only 4.8 × 10^−6^% at a single-stage yield of 90% (Fig. 4f and g). However, if the separation factor could be enhanced to 0.8 or 0.5, the separation stages would be reduced sharply to 35 and 12, respectively. With concomitant improvements in single-stage yield to 95% and 97.5%, the total yield could reach a high level of 17% and 74%.

According to eqn (5) and (6), two general formulae can be further derived for the number of separation stages required at the anode (*n*_anode_) and the cathode side (*n*_cathode_) to obtain ^32^S with an abundance >99.9% and ^34^S with an abundance >99%:

7 *n*_anode_ = ⌈ln(6660/149)/ln(*α*_anode_)⌉

8 *n*_cathode_ = ⌈−ln(2212.65)/ln(*α*_cathode_)⌉

where in the ceiling function ⌈⌉ denotes rounding up to the nearest integer. Based on eqn (7) and (8), the overall yield at the anode and the cathode respectively can be expressed as

9 *Y*^all^_anode_ = *Y*_anode_^*n*(anode)^ = *Y*_anode_^⌈ln(6660/149)/ln(*α*_a_)⌉^

10 *Y*^all^_cathode_ = *Y*_cathode_^*n*(cathode)^ = *Y*_cathode_^⌈−ln(2212.65)/ln(*α*_c_)⌉^

According to eqn (9) and (10), 3D plots illustrating the correlation between the overall yield, single-stage yield, and separation factor are presented in Fig. S9a and b. As expected, higher single-stage yields and separation factors consistently enhance the overall yield for both ^32^S at the anode and ^34^S at the cathode. In practical electrochemical isotope separation scenarios, a critical concern is whether highly efficient separation can be simultaneously achieved for both ^32^S and ^34^S within a single Li–S cell. As mentioned above, the separation factors and single-stage yields of ^32^S and ^34^S are intrinsically coupled by the law of isotopic mass conservation (eqn (1)–(3)). Consequently, the separation efficiency of ^32^S inherently determines that of ^34^S, and *vice versa*. By substituting eqn (1)–(3) into eqn (9) and (10), we can quantify the mutual influence between the separation efficiencies of ^32^S and ^34^S, as depicted in Fig. S9c and d:

11 *Y*^all^_anode_ = (1 − *Y*_cathode_)^⌈ln(6660/149)/ln((1 + *α*_c_ − 2*α*_c_*Y*_c_)/(1 + *α*_c_ − 2*Y*_c_))⌉^

12 *Y*^all^_cathode_ = (1 − *Y*_anode_)^⌈−ln(2212.65)/ln(*α*_a_(2*Y*_a_ − 1) − 1]/[(2*Y*_a_ − 1) − *α*_a_)⌉^

**Fig. 5:**
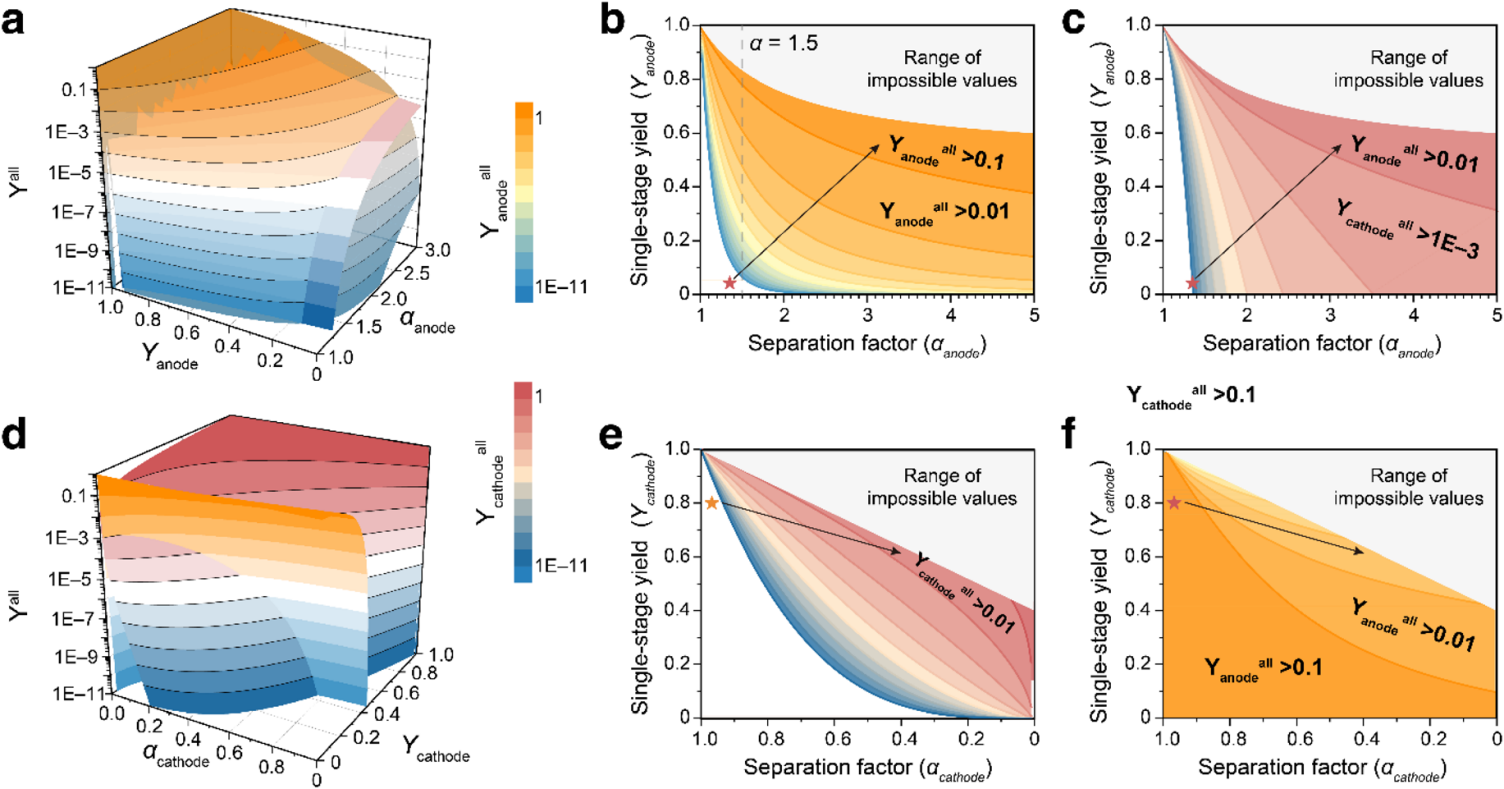
(a) 3D surface plot showing the synchronous growth of overall yields for both ^32^S and ^34^S as a function of anode separation factor (*α*_anode_) and single-stage yield (*Y*_anode_). (b and c) Contour plots illustrating the efficiency gradients for (b) ^32^S (blue-yellow gradient) and (c) ^34^S (blue-red gradient) as a function of anode-side separation parameters. (d) 3D surface plot revealing an inverse relationship between ^32^S and ^34^S overall yields as a function of anode separation factor (*α*_cathode_) and single-stage yield (*Y*_cathode_). (e and f) Contour plots demonstrating the efficiency gradients for (e) ^34^S and (f) ^32^S as a function of cathode-side parameters.

The corollaries of isotopic mass conservation provide a roadmap for optimizing separation parameters to maximize isotope separation performance. Fig. 5a–f summarize the coupled effects of anode-side and cathode-side separation parameters on the separation efficiencies of ^32^S and ^32^S, respectively, where the blue-yellow and blue-red color scales denote the overall yield gradients for ^32^S and ^34^S. As shown in Fig. 5a, the overall yields of both ^32^S and ^34^S change synchronously while tuning anode-side separation parameters; *i.e.*, enhancing the anode separation factor (*α*_anode_) and single-stage efficiency (*Y*_anode_) not only boosts the overall yield of ^32^S at the anode, but also improves the ^34^S yield at the cathode. The contour plots in Fig. 5b and c reveal that when the overall yield of ^32^S is increased from the current stage to >10%, the overall yield of ^34^S at the cathode side concurrently rises to >1%. By contrast, according to Fig. 5d, adjustment of the cathode-side separation parameters leads to an inverse relationship between the overall yields of ^32^S and ^34^S. Specifically, enhancing the cathode separation factor (*α*_cathode_) or single-stage efficiency (*Y*_cathode_) will, in most cases, sacrifice the overall yield of ^32^S.

For ^32^S enrichment, Fig. 5b suggests that increasing the separation factor is an effective strategy at a relatively low anode separation factor (*e.g. α*_anode_ <1.5). However, at substantially higher separation factors (*e.g. α*_anode_ >3), simultaneous improvement of both the separation factor and the single-stage yield is required to further enhance overall efficiency. For ^34^S enrichment, according to Fig. 5e, given the currently high single-stage yield at the cathode side, the critical challenge for achieving efficient ^34^S separation is to balance the separation factor and the single-stage yield to obtain optimal separation efficiency.

Generally, a higher separation factor and single-stage yield both contribute to improved separation efficiency. Despite the high separation factor of ^32^S at the anode side, the overall yield remains extremely low as a result of low single-stage yield. For the separation of ^34^S at the cathode side, the current primary constraint is the low separation factor, which necessitates numerous separation stages (151 stages at *α* = 0.95). However, constrained by the law of isotopic mass conservation (eqn (1)–(3)), it is challenging to simultaneously achieve high-efficiency separation of both ^32^S and ^34^S in a Li–S battery. Based on the preceding discussions, prioritizing improvements in the separation factor and single-stage yield for ^32^S at the anode side is generally more advantageous, as it concurrently bolsters the separation efficiency of ^34^S, thereby adding to the overall separation benefits. In other words, Li–S batteries represent a more proficient platform for the separation and recovery of ^32^S compared to ^34^S. In practical applications, the cell parameters, material compositions, and (dis)charge protocols could be further regulated to achieve optimal separation performance.

**Fig. 6:**
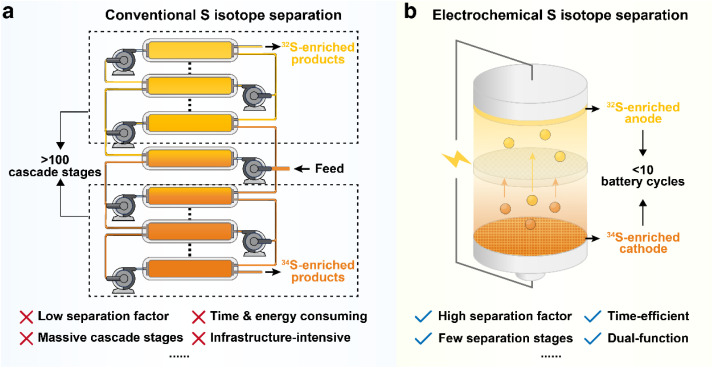
Comparison between (a) conventional S isotope separation techniques and (b) the electrochemical isotope separation method.

Compared to conventional S isotope separation techniques, such as chemical exchange, thermal diffusion, and cryogenic distillation, electrochemical isotope separation in a Li–S battery offers comprehensive advantages (Fig. 6). Conventional methods are typically hindered by limited separation factors (usually <40‰), which necessitates massive, complex cascade systems and prolonged operation times to achieve significant enrichment, leading to exorbitant energy consumption and the requirement for large-scale specialized infrastructure.^28–30^ In stark contrast, electrochemical isotope separation utilizing Li–S batteries achieves remarkably higher separation factors, which drastically reduces the required number of separation stages and shortens the processing duration. Based on the current separation factors (*e.g. α* = 1.5), ^32^S with an isotope abundance exceeding 99.9% can be enriched from natural S within just ten cascading stages.

More importantly, electrochemical methods enable efficient fractionation of S isotopes during the (dis)charging cycles of Li–S batteries, which effectively transforms a standard energy storage device into a dual-purpose platform that yields high value-added isotopic products. By leveraging the simple, scalable Li–S battery configurations and the infrastructure of the rapidly growing energy storage industry, electrochemical isotope separation significantly reduces equipment dependence and operational complexity. The law of isotopic mass conservation provides the theoretical guidance for maximizing the efficiency of S isotope separation. These advantages position electrochemical isotope separation in Li–S batteries as a highly promising and sustainable roadmap for future isotope enrichment technologies.

## Conclusions

In summary, we established a standard protocol for quantitative analysis of abundance and ratios of S isotopes using TQ-ICP-MS. Based on this method, we have, for the first time, verified the mass conservation of S isotopes in a Li–S battery, wherein electrochemical cycling consistently enriched ^34^S in the cathode and ^32^S in the anode and electrolyte. The mass conservation holds consistently across different states of charge of the battery. Beyond experimental verification, isotopic mass conservation allows us to establish a quantitative relationship between the separation efficiencies of ^32^S and ^34^S. The separation factors at two electrodes are intrinsically coupled and unfavorable trade-offs exist between the separation factor and the single-stage yield, as well as between the yields of the two isotopes. While the electrochemical separation of ^32^S is limited by a low single-stage yield and that of ^34^S by a modest separation factor, simultaneous improvements in these parameters could help increase the separation efficiency. This work not only validates the kinetic isotope effects during battery operation as a viable mechanism for S isotope separation, but also provides a quantitative framework for evaluating and optimizing the electrochemical isotope separation strategies. By clarifying the governing constraints imposed by isotopic mass conservation, our results offer guidance for rational design of next-generation advanced electrochemical systems for isotope separation.

## Author contributions

Yu-Hui Zhu: conceptualization, data curation, methodology, investigation, formal analysis, visualization, writing – original draft and writing – review & editing. Sen Xin: conceptualization, project administration, supervision, investigation, validation, resources, funding acquisition and writing – review & editing.

## Conflicts of interest

There are no conflicts to declare.

## Supplementary Material

SC-017-D5SC10219G-s001

## Data Availability

The data supporting this article have been included as part of the supplementary information (SI). Other data that support the findings of this study are available from the corresponding author upon reasonable request. Supplementary information: experimental procedures, supporting characterization techniques and mathematical analysis, and details for TQ-ICP-MS results. See DOI: https://doi.org/10.1039/d5sc10219g.
