# Supplementary material for: Electrochemical fractionation of stable sulfur isotopes in a rechargeable lithium–sulfur battery: a revisit from the law of mass conservation
Source: Chem Sci. 2026 Feb 14;17(14):7255–64. doi: 10.1039/d5sc10219g (PMC12915562; doi:10.1039/d5sc10219g)
Supplement: SC-017-D5SC10219G-s001 [file SC-017-D5SC10219G-s001.pdf]

Supplementary Information (SI) for Chemical Science.
This journal is © The Royal Society of Chemistry 2026

Supplementary Information for

Electrochemical fractionation of stable sulfur isotopes in a rechargeable lithium-sulfur battery: a revisit from the law of mass conservation

Yu-Hui Zhu[*a, b] and Sen Xin*[*a, b]*

[a] *Laboratory of Molecular Nanostructure and Nanotechnology, CAS Research/Education Center for Excellence in Molecular Sciences, Beijing National Laboratory for Molecular Sciences (BNLMS), Institute of Chemistry, Chinese Academy of Sciences (CAS), Beijing 100190, P. R. China. Email: zhuyuhui@iccas.ac.cn; xinsen08@iccas.ac.cn*

[b] *School of Chemical Sciences, University of Chinese Academy of Sciences, Beijing 100049, P. R. China.*

Experimental Procedures

Chemicals and reagents. *α*-^{32}S (natural S, analytical reagent), HNO_3 (70%), anhydrous NaOH (≥98%), 1,2-dimethoxyethane (DME), 1,3-dioxolane (DOL) and $LiPF_6$ were purchased from Sigma-Aldrich. H_2O_2 (30%) was purchased from Sinopharm Chemical Reagent Co., Ltd. *α*-^{34}S (99.79%) was purchased from Cambridge Isotope Laboratories, Inc. CMK-3 mesoporous carbon was purchased from Nanjing XFNANO Materials. Carbon-coated Al foil was purchased from Guangzhou Nanuo New Materials Technology Co., Ltd. Super-P was purchased from Cabot. Poly (vinylidene fluoride) (PVDF) and 1-methyl-2-pyrolidone (NMP) were purchased from Alfa Aesar. Li metal was purchased from Tianjin Zhongneng Lithium Co. The reference sample of S isotope for ICP-MS analysis was purchased from National Institute of Metrology, China.

Preparation of $^{32}S/^{34}S$@CMK-3 electrod*e*. The $^{32}S/^{34}S$@CMK-3 composite was prepared by mixing *α*-^{32}S and *α*-^{34}S powders and CMK-3 thoroughly in an agate mortar at a mass ratio of 194:206:600, and heating the mixture in an Ar-prefilled container at 155 ^{o}C for 12 h. The $^{32}S/^{34}S$@CMK-3 composite was prepared after the mixture was cooled down to room temperature. The $^{32}S/^{34}S$@CMK-3 electrode was prepared by mixing the as-prepared $^{32}S/^{34}S$@CMK-3 composite, super-P conductive additives and PVDF at a mass ratio of 8:1:1 in NMP to form a homogeneous slurry. The slurry was casted onto a carbon-coated Al foil and dried at 70 ^{o}C for 12 h. The dried foils were cut into electrode slices with a diameter of 10 mm with an areal mass loading of 0.8 mg_S/cm^2.

Cell fabrication and electrochemical measurements. Li-S batteries were assembled using the Swagelok-type cell in an Ar-filled glove box (Mikrouna, with H_2O and O_2 contents of <0.01 ppm). The electrolyte was prepared by dissolving 1M $LiPF_6$ in the mixture of DME and DOL (v:v = 4:1). $^{32}S/^{34}S$@CMK-3 electrode was paired with a Li-metal electrode. Glass fiber films (Whatman, GF/F, 10 mm diameter) were used as the separator. 150 μL of electrolyte with three GF/F films were placed between the cathode and Li metal. The assembled cells were rested for 4 hours and then transferred to a temperature-controlled chamber at 25 ^{o}C for electrochemical testing. The galvanostatic discharge and charge tests were carried out on a LAND system in voltage ranges of 1.5 V to 2.8 V under 25 ^{o}C.

Sample preparation for ICP-MS tests. The cycled Swagelok-type cell was

disassembled in the Ar-filled glove box to separate cathode, anode and separator. The cycled Li and cathode were washed by DME for three times to remove the electrolyte. The cycled cathode was added into 70% HNO_3 and heated at 60 °C. After complete oxidation, the solution is diluted to *c.a.* 10 μmol_S L^{-1} with deionized water prior to test. The cycled Li anode and separator was dissolved in 0.01 M NaOH solution. For the direct measurement method, the resulting solution was directly diluted to *c.a.* 10 μmol_S L^{-1} with 0.01 M NaOH solution prior to test. For the post-oxidation method, the resulting solution was added by 30% H_2O_2 and held at 60 °C to fully oxidize S^{2-} and S_x^{2-} to SO_4^{2-}. The solution was then calibrated to a fixed volume and diluted to *c.a.* 10 μmol_S L^{-1} with deionized water and dilute HNO_3 solution prior to test.

Materials characterization. Thermogravimetric profiles were collected on a Netzsch thermal gravimetric analyzer (TG/DTA6300) from room temperature to 800 °C at a heating rate of 10 °C/min under the N_2 atmosphere. XPS measurements were conducted on Thermo Scientific ESCALab 250Xi (Thermo Fisher Scientific). The *in situ* Raman spectra were collected on a on a HORIBA spectrometer (LabRAM HR Evolution) with a 10× objective and 532-nm excitation laser. The isotopic abundance and ratio of ^{32}S and ^{34}S was examined by an ICP-MS (Agilent 8900). TOF-SIMS experiments were performed on a TOF-SIMS spectrometer (ION-TOF GmbH, Münster, Germany) equipped with a 30 keV Bi^{3+} primary ion gun and a 2-keV Cs^+ sputter gun.

Note S1

Isotope separation factor is used to quantify the change in the ratio of two isotopes before and after separation. It is defined as the ratio of the isotope abundance ratio in the product phase to that in the initial phase and can be expressed as:

$$\alpha = (X_P/Y_P)/(X_R/Y_R) \qquad \text{(S1)}$$

where α is the separation factor, X_P and Y_P denote the molar fractions of the two isotopes in the products, and X_R and Y_R denote their molar fractions in reactants.

As the separation factors are typically close to unity, the separation factor is often expressed in per mil notation to facilitate comparison, which can also be defined as:

$$\delta = (\alpha\text{-}1)\times 1000 \qquad \text{(S2)}$$

Note S2

Assume the total molar amount of in the pristine cathode is n, and initial ratio of ^{32}S:^{34}S is 1:1. Then ^{32}S and ^{34}S both account for $n/2$.

For a cycled Li-S battery, the molar amount of ^{32}S and ^{34}S in the cathode are denoted as n_1^2 and n_1^4; and the molar amount of ^{32}S and ^{34}S in the anode and electrolyte are denoted as n_2^2 and n_2^4. The separation factor (α_1) and yield (Y_1) for the cathode can be expressed as:

$$\alpha_1 = n_1^2/n_1^4 \tag{S3}$$

$$Y_1 = (n_1^2+n_1^4)/n \tag{S4}$$

Similarly, the separation factor (α_2) and yield (Y_2) for the anode and electrolyte can be expressed as:

$$\alpha_2 = n_2^2/n_2^4 \tag{S5}$$

$$Y_2 = (n_2^2+n_2^4)/n \tag{S6}$$

Based on equation 3 to 6, we can derive:

$$n_1^2 = \alpha_1 n Y_1/(\alpha_1+1) \tag{S7}$$

$$n_2^2 = \alpha_2 n Y_2/(\alpha_2+1) \tag{S8}$$

$$Y_1 + Y_2 = 1 \tag{S9}$$

Equation 9 represents mass conservation of S in a Li-S battery. According to the isotopic conservation of S:

$$n_1^2 + n_2^2 = n/2 \tag{S10}$$

$$n_1^4 + n_2^4 = n/2 \tag{S11}$$

By substituting equations 7 and 8 into equations 10 and 11, respectively, we obtain

$$\alpha_1 Y_1/(\alpha_1+1) + \alpha_2 Y_1/(\alpha_2+1) = 1/2 \tag{S12}$$

$$Y_1/(\alpha_1+1) + Y_1/(\alpha_2+1) = 1/2 \tag{S13}$$

Equations 12 and 13 can be further simplified to:

$$Y_1 = (\alpha_1+1)(\alpha_2-1)/[2(\alpha_2-\alpha_1)] \tag{S14}$$

$$Y_2 = (1-\alpha_1)(\alpha_2+1)/[2(\alpha_2-\alpha_1)] \tag{S15}$$

$$\alpha_1 = \alpha_2[(2Y_2-1)-1]/[(2Y_2-1) -\alpha_2] \tag{S16}$$

Note S3

For any constant $0<\alpha_1<1$, by taking the first-order derivative of equation 2, we obtain:

$$dY_1/d\alpha_2 = (1-\alpha_1^2)/[2(\alpha_2-\alpha_1)^2] > 0 \tag{S17}$$

That is to say, for any $0<\alpha_1<1$, Y_1 decreases as α_2 decreases. Under varying conditions of $0<\alpha_1<1$, the range of minimum values for Y_1 is consistently greater than the value obtained at α_2=1, and the maximum values for Y_1 can be approached at an infinitely high α_2; thus, we have:

$$Y_1 > (\alpha_1+1)(1-1)/[2(1-\alpha_1)] = 1/2 + 1/(2\alpha_1) = 0 \tag{S18}$$

$$Y_1 < (\alpha_1+1)(\alpha_2-1)/[2(\alpha_2-\alpha_1)] \approx \alpha_1/2 + 1/2 \tag{S19}$$

Therefore, the allowable ranges for α_1 and Y_1 are as follows:

$$0 < Y_1 < \alpha_1/2 + 1/2 \tag{S20}$$

Likewise, for any constant $\alpha_2>1$, by taking the first-order derivative of equation S15, we obtain:

$$dY_2/d\alpha_1 = (1-\alpha_2^2)/[2(\alpha_2-\alpha_1)^2] < 0 \tag{S21}$$

for any $\alpha_2>1$, Y_2 increases as α_1 decreases. The maximum value of Y_2 is reached at α_1=0. Under varying conditions of $\alpha_2>1$, the range of maximum values for Y_2 is consistently smaller than the value obtained at α_1=0; thus, we have:

$$Y_2 < (1-0)(\alpha_2+1)/[2(\alpha_2-0)] = 1/2 + 1/(2\alpha_2) \tag{S22}$$

and the minimum values for Y_2 can be approached when α_1 approaches 1:

$$Y_2 > (1-1)(\alpha_2+1)/[2(\alpha_2-1)] = 0 \tag{S23}$$

The allowable ranges for α_2 and Y_2 are as follows:

$$0 < Y_1 < 1/2 + 1/(2\alpha_2) \tag{S24}$$

Supplementary Figures and Tables

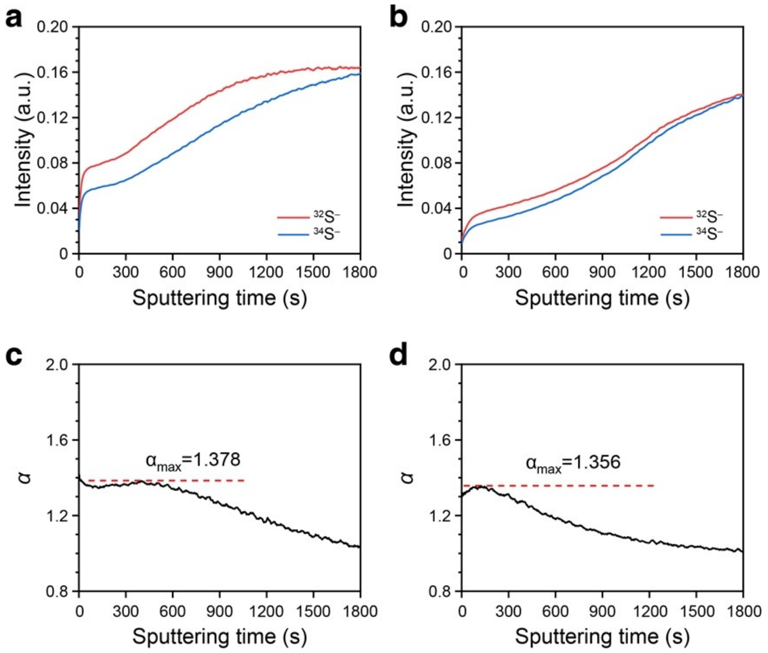

Figure S1. (a-b) Normalized $^{32}S^{-}$ and $^{34}S^{-}$ intensities of ToF-SIMS results at two different sampling regions of the same Li anode from a cycled Li-S battery and (c-d) corresponding separation factors (α).

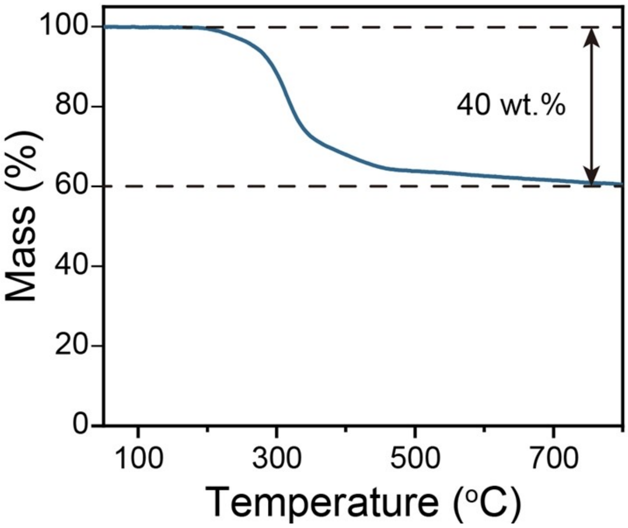

Figure S2. Thermogravimetric profiles of the composite S/C material.

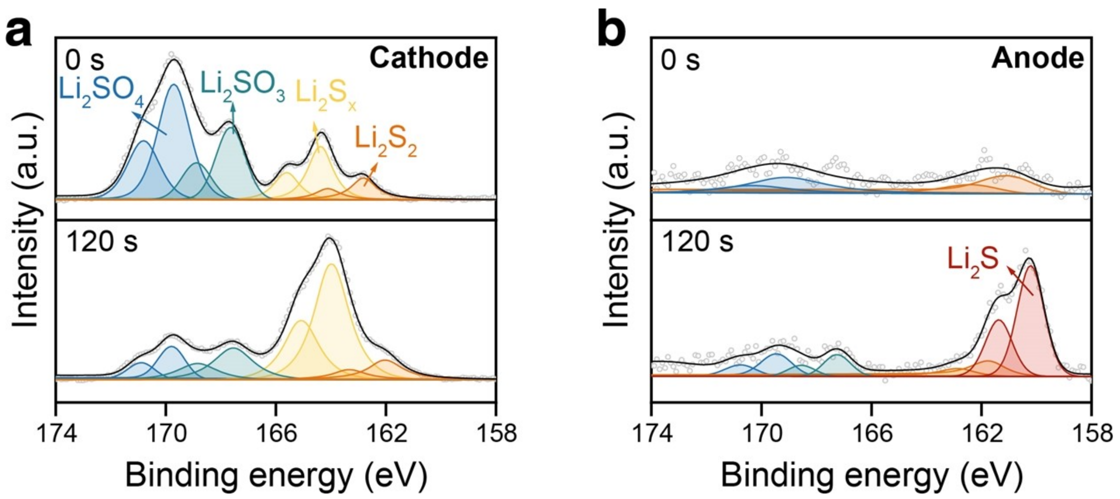

Figure S3. S 2p XPS spectra of the (a) cathode and (b) anode of a Li-S battery being discharged to 1.5 V.

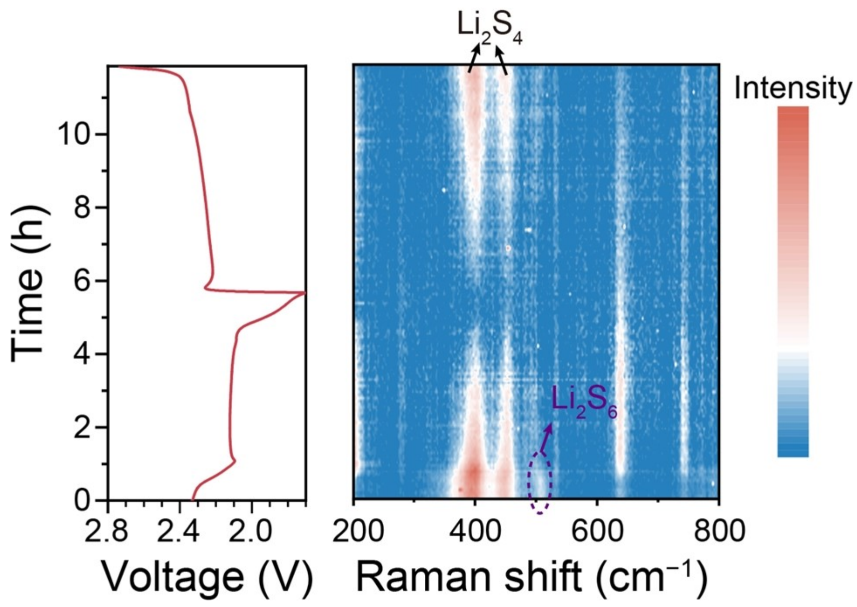

Figure S4. In-situ Raman spectra of a Li-^{32}S battery.

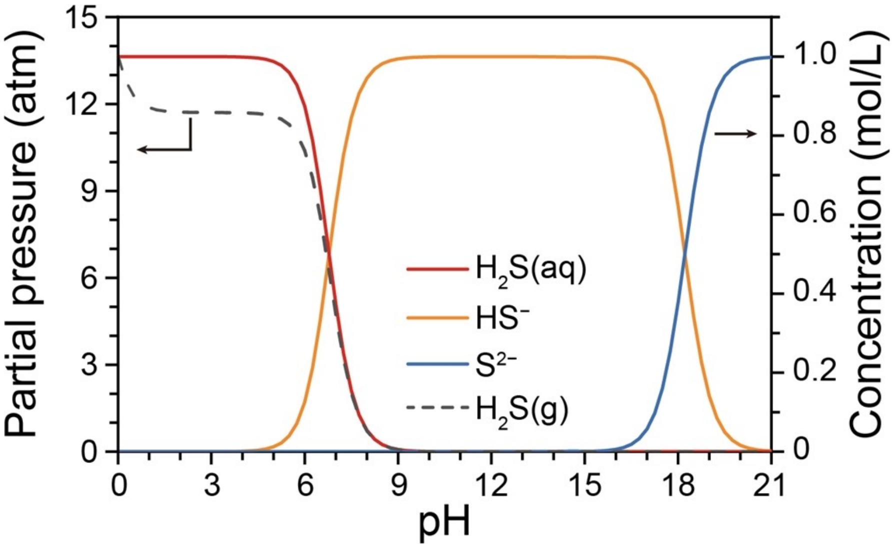

Figure S5. Chemical equilibrium of S^{2-}, HS^{-} and H_2S at a concentration of 1 mol/L S.

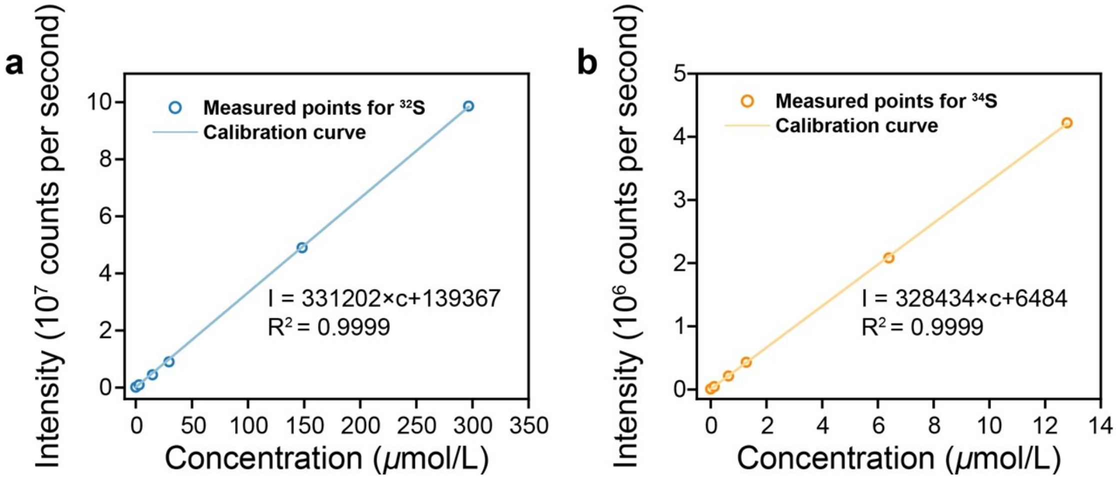

Figure S6. Calibration curves by TQ-ICP-MS for (a) ^{32}S, concentration range: 2.965 μmol L^{-1} to 296.5 μmol L^{-1}; and (b) ^{34}S, concentration range: 0.1279 μmol L^{-1} to 12.79 μmol L^{-1}.

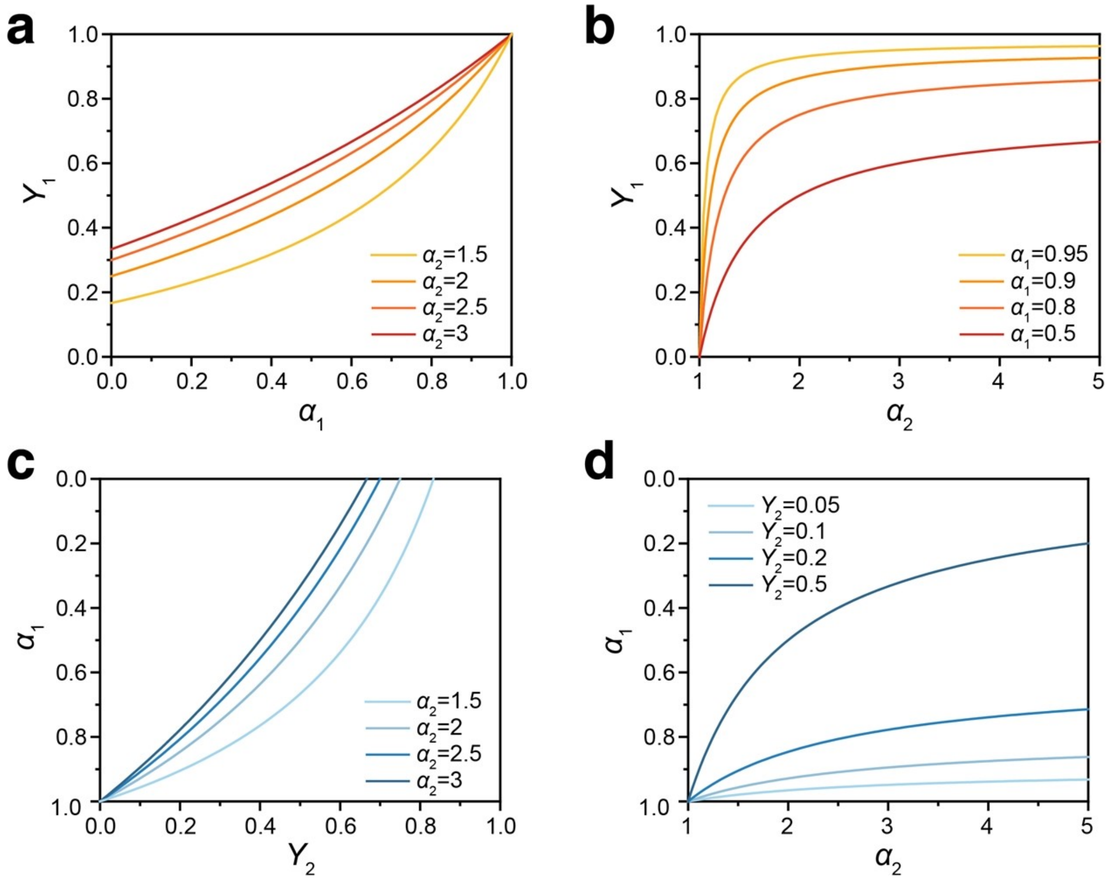

Figure S7. (a) Relationship between ^{34}S yield (Y_1) and ^{34}S separation factor (α_1) at fixed ^{32}S separation factors (α_2). (b) Relationship between Y_1 and α_2 at fixed α_1. (c) Relationship between α_1 and ^{32}S yield (Y_2) at fixed α_2. (d) Relationship between α_1 and α_2 at fixed Y_2.

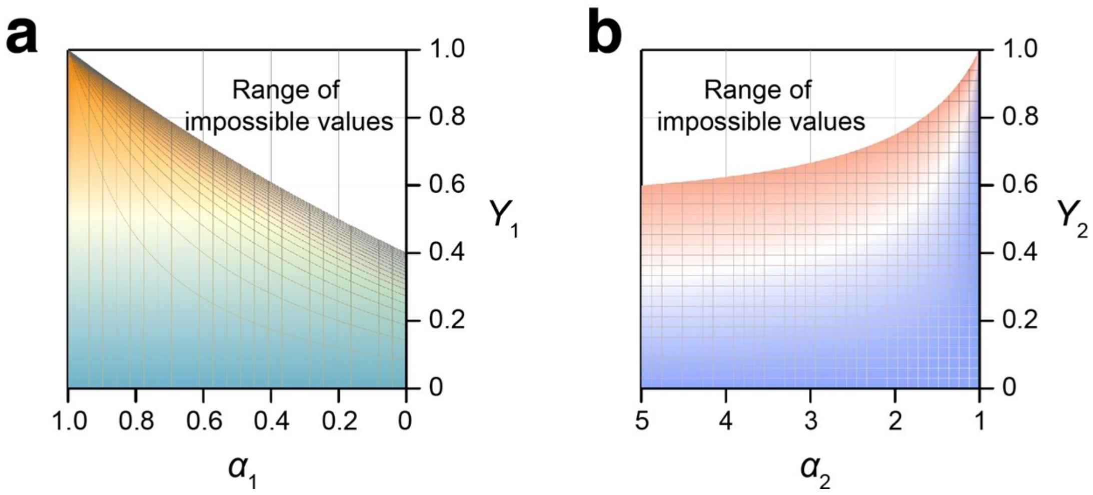

Figure S8. (a) Projections of Fig. 4b onto the α_2 axis and (b) Fig. 4c onto the α_1 axis.

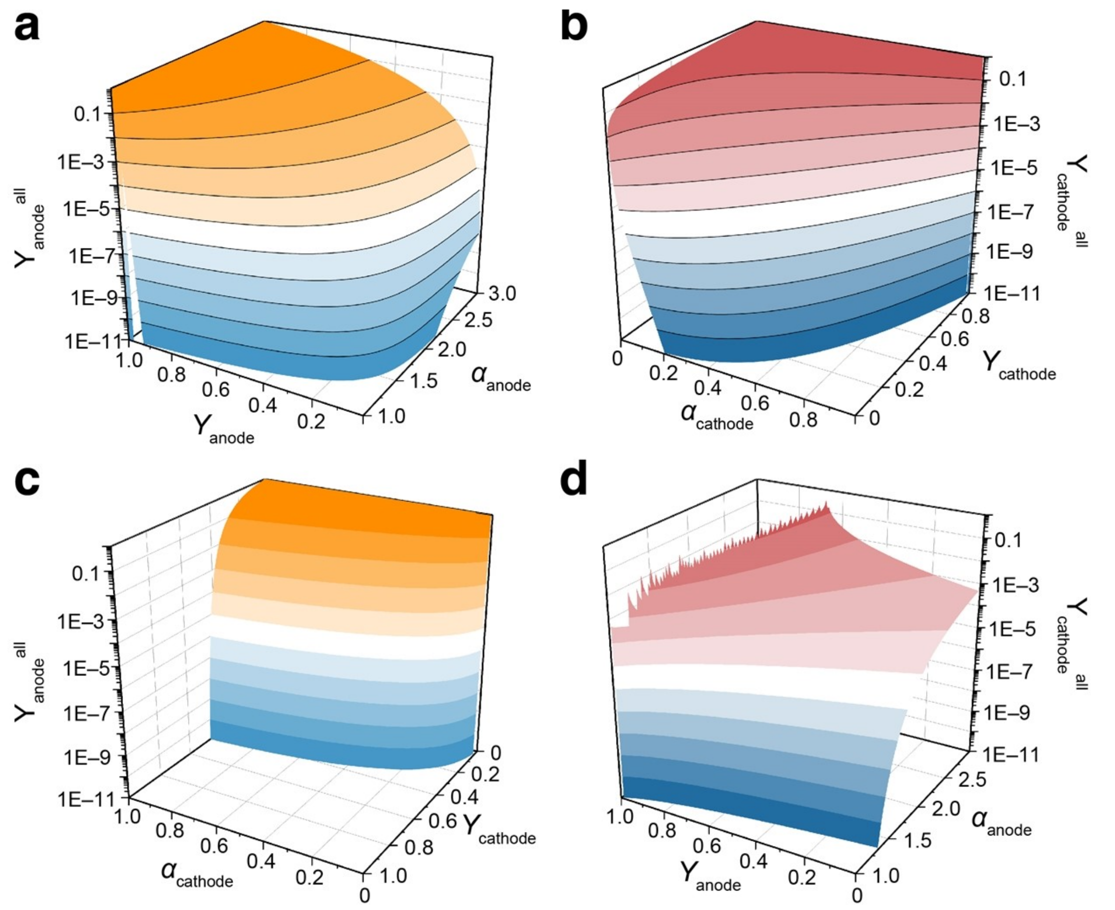

Figure S9. (a) Relationship between overall yield of ^{32}S at anode side ($Y_{\mathrm{anode}}^{\mathrm{all}}$) and ^{32}S separation factor (α_{anode}) and ^{32}S single-stage yield (Y_{anode}). (b) Relationship between overall yield of ^{34}S at cathode side ($Y_{\mathrm{cathode}}^{\mathrm{all}}$) and ^{34}S separation factor ($\alpha_{\mathrm{cathode}}$) and ^{34}S single-stage yield (Y_{cathode}). (c) Relationship between overall yield of ^{32}S at anode side ($Y_{\mathrm{anode}}^{\mathrm{all}}$) and ^{34}S separation factor ($\alpha_{\mathrm{cathode}}$) and ^{34}S single-stage yield (Y_{cathode}). (d) Relationship between overall yield of ^{344}S at anode side ($Y_{\mathrm{cathode}}^{\mathrm{all}}$) and ^{32}S separation factor (α_{anode}) and ^{32}S single-stage yield (Y_{anode}).

Table S1. Isobaric and polyatomic interferences on S isotope masses by conventional ICP-MS.

Isotope	Nominal mass	Spectral interference	Resolution (Δm/m)
^{32}S	32	$^{16}O^{16}O^+$	1801
		$^{14}N^{18}O^+$	1061
		$^{15}N^{16}OH^+$	1040
		Zn^{2+}	–4562
^{34}S	34	$^{16}O^{18}O^+$	1297
		$^{16}O^{17}OH^+$	1000
		$^{16}O^{16}OH_2^+$	904
		$^{33}SH^+$	2977

Table S2. Key TQ-ICP-MS parameters for S isotope measurement.

**Parameters**	**Value**
RF power (W)	1550
Sample depth (mm)	8
Nebulizer gas flow rate (L/min)	1.03
Extraction lens 1 (V)	–4
Extraction lens 2 (V)	–210
Omega bias (V)	–100
Omega (V)	10
Cell gas mode	O_2
Cell gas flow (mL/min)	0.45
Load time (s)	60
Stabilization time (s)	30
Rinse time (s)	30

Table S3. TQ-ICP-MS results for total S mass recovery of Li_2S and S/C composites.

**Sample**	Li_2S (direct measurement)	Li_2S (post-oxidation measurement)	C/S composite
**^{32}S (CPS*)**	1.735×10^6	4.102×10^6	9.385×10^5
**^{32}S (μmol L^{-1})**	4.583	10.84	2.413
**^{34}S (CPS)**	8.558×10^4	1.934×10^5	8.813×10^5
**^{34}S (μmol L^{-1})**	0.210	0.475	2.662
**Total mass (mg)**	3.076	7.261	5.367
**Standard mass (mg)**	3.10	7.38	5.44 (mass of S) 13.60 (mass of S/C composite)
**Mass fraction (%)**	99.2%	98.4%	98.7%

*CPS refers to counts per second.

Table S4. TQ-ICP-MS results for blank solution tests.

**Sample**	**^{32}S (CPS)**	**^{32}S (μmol L^{-1})**	**^{34}S (CPS)**	**^{34}S (μmol L^{-1})**
Test for direct measurement	1.735×10^{6}	4.583	8.558×10^{4}	0.210
Blank test	7.499×10^{4}	0.195	1.964×10^{4}	0.041
Test for post-oxidation measurement	7.695×10^{6}	22.81	7.857×10^{6}	23.90
Blank test	5.69×10^{4}	<0	3.044×10^{3}	<0

Table S5. TQ-ICP-MS results of ^{32}S and ^{34}S for the anode of cycled Li-S batteries.

**Sample (Anode)**	**^{32}S (CPS)**	**^{32}S (μmol L^{-1})**	**^{34}S (CPS)**	**^{34}S (μmol L^{-1})**	**$^{32}S/^{34}S$**	α
Pristine	9.385×10^5	2.413	8.813×10^5	2.662	0.9065	
C-2.20V	6.889×10^5	1.659	4.411×10^5	1.322	1.255	1.384
C-2.38V	1.148×10^6	3.044	8.090×10^5	2.442	1.247	1.375
1 cycle	1.291×10^6	3.479	9.475×10^5	2.864	1.215	1.340
2 cycles	2.288×10^6	6.488	2.158×10^6	6.550	0.9905	1.093

Table S6. TQ-ICP-MS results of ^{32}S and ^{34}S for the cathode of cycled Li-S batteries.

**Sample (Cathode)**	**^{32}S (CPS)**	**^{32}S (μmol L^{-1})**	**^{34}S (CPS)**	**^{34}S (μmol L^{-1})**	**$^{32}S/^{34}S$**	**α**
Pristine	9.385×10^5	2.413	8.813×10^5	2.662	0.9065	
C-2.20V	1.679×10^6	4.650	1.718×10^6	5.211	0.8923	0.984
C-2.38V	1.553×10^6	4.270	1.609×10^6	4.877	0.8755	0.966
1 cycle	1.812×10^6	5.052	1.889×10^6	5.731	0.8815	0.972
2 cycles	1.856×10^6	5.182	1.930×10^6	5.857	0.8847	0.976

Table S7. TQ-ICP-MS results of ^{32}S and ^{34}S for the electrolyte of cycled Li-S batteries.

**Sample (electrolyte)**	**^{32}S (CPS)**	**^{32}S (μmol L^{-1})**	**^{34}S (CPS)**	**^{34}S (μmol L^{-1})**	**$^{32}S/^{34}S$**	**α**
Pristine	9.385×10^5	2.413	8.813×10^5	2.662	0.9065	
C-2.20V	3.488×10^6	10.111	3.375×10^6	10.255	0.9859	1.088
C-2.38V	2.927×10^6	8.417	2.874×10^6	8.729	0.9643	1.064
1 cycle	2.486×10^6	7.084	2.398×10^6	7.280	0.9731	1.073
2 cycles	2.235×10^6	6.326	2.139×10^6	6.491	0.9746	1.075

Table S8. TQ-ICP-MS results for the verification of S isotope mass conservation.

**Sample**		**C-2.20V-1**	**C-2.20V-2**	**C-2.38V-1**	**C-2.38V-2**	**1 cycle-1**	**1 cycle-2**	**2 cycles-1**	**2 cycles-2**
**Anode**	**^{32}S (μg)**	5.309	4.970	9.741	8.198	11.13	6.240	20.76	13.73
	**^{34}S (μg)**	4.495	4.277	8.303	7.402	9.738	5.576	22.27	14.63
	**Total (μg)**	9.804	9.247	18.04	15.60	20.87	11.82	43.03	28.36
**Cathode**	**^{32}S (mg)**	0.2232	0.2872	0.2050	0.1776	0.2425	0.2134	0.2487	0.3151
	**^{34}S (mg)**	0.2658	0.3425	0.2487	0.2128	0.2923	0.2490	0.2987	0.3794
	**Total (mg)**	0.4890	0.6297	0.4537	0.3904	0.5348	0.4624	0.5474	0.6945
**Electro-lyte**	**^{32}S (mg)**	0.07765	0.03818	0.06464	0.04737	0.05441	0.06139	0.04859	0.06298
	**^{34}S (mg)**	0.08368	0.03980	0.07123	0.05182	0.05941	0.06695	0.05297	0.07011
	**Total (mg)**	0.1613	0.07798	0.1359	0.09919	0.1138	0.1283	0.1016	0.1331
**Total mass (mg)**		0.6602	0.7170	0.6076	0.5052	0.6695	0.6025	0.6920	0.8560
**Pristine (mg)**		0.6720	0.7392	0.6592	0.5440	0.7104	0.6336	0.7072	0.8768
**Mass fraction (%)**		98.24	97.00	92.17	92.87	94.24	95.09	97.85	97.63
**Average mass fraction (%)**		95.64%							
**Total mole of ^{32}S (μmol)**		9.568	9.598	8.729	7.288	9.626	8.781	9.940	12.24
**Total mole of ^{34}S (μmol)**		10.41	10.56	9.654	8.000	10.63	9.458	11.00	13.65
**^{32}S/^{34}S**		0.9191	0.9089	0.9042	0.9110	0.9055	0.9284	0.9036	0.8967
**Average ^{32}S/^{34}S**		0.9097							
**Pristine**		0.9065							
